# Usability and Quality of the JoyPop App: Prospective Evaluation Study

**DOI:** 10.2196/65472

**Published:** 2025-07-04

**Authors:** Ishaq Malik, Teagan Neufeld, Aislin Mushquash

**Affiliations:** 1 Department of Psychology, Lakehead University, 955 Oliver Road, Thunder Bay, ON, P7B 5E1, Canada, 1 8073438010 ext 8771

**Keywords:** usability, quality, mobile health, mental health app, postsecondary students, uMARS, MAUQ, evaluation study, mental health, app, resilience, Canada, student, usefulness, satisfaction, regression analyses, coping, smartphone app, mHealth app, mHealth, users, user version of the Mobile Application Rating Scale, Mobile Application Usability Questionnaire

## Abstract

**Background:**

Mental health difficulties are increasing among Canadian postsecondary students, and many face barriers to accessing mental health care. Mobile health smartphone apps for mental health reduce common barriers to care and improve student mental health outcomes. However, students’ engagement and use of mental health apps is low. Evaluating the usability and quality of mental health apps is essential not only for user engagement but also for safety and overall utility. Few mental health apps have undergone usability and quality evaluations, especially with measures explicitly designed for these apps. The JoyPop app is a resilience-building mental health app with evidence supporting its effectiveness for student mental health. It has yet to be evaluated using standardized measures of mental health app usability and quality, and the influence of usability and quality on use is unknown.

**Objective:**

We evaluated the usability and quality of the JoyPop app and the predictive importance of usability and quality, compared to other relevant user characteristics, in predicting intentions to use the app in the future (usage intentions).

**Methods:**

Participants (N=183) completed preapp measures assessing demographics and personality traits, then used the app for 1 week, and then completed postapp measures assessing the usability, quality, and use of the JoyPop app. Usability (overall; and subscales: ease of use, interface and satisfaction, and usefulness) and quality (objective, subjective, and perceived impact) were assessed with descriptive statistics. Multiple regression analyses tested the predictive importance of usability and quality on usage intentions after controlling for other user characteristics.

**Results:**

Participants rated the JoyPop app’s overall usability as “very good” (mean 5.63, SD 0.85). Participants rated the JoyPop app’s overall objective quality as “excellent” (mean 4.06, SD 0.54). Subjective quality ratings were good, with many participants (135/183, 73.8%) indicating they would recommend the app to others. Participants rated the app as having a moderate and helpful impact on their mental health and coping skills (mean 3.48, SD 0.88). In each regression model, usability (β=.56, *P*<.001) and quality (β=.52, *P*<.001) were the strongest predictors and predicted usage intentions over and above other user characteristics.

**Conclusions:**

Results align with prior research evaluating the JoyPop app and maintain that it is an engaging and high-quality mental health app that can support students. Findings provide important insight into the optimal design of mental health apps for students and inform adaptations to future iterations of the JoyPop app.

## Introduction

### Background

Mental health difficulties among Canadian postsecondary students are increasing [1-3]. Unaddressed mental health difficulties can exacerbate existing challenges and negatively impact academic and long-term health outcomes [4,5]. Concerningly, students face a multitude of barriers (eg, long wait times and financial constraints) to access mental health support [4,6], resulting in prolonged distress, difficulties for campus mental health services, and low future help-seeking behaviors [7,8]. Novel approaches that increase students’ access to mental health support are needed [9,10].

Mobile health smartphone apps designed to improve mental health are promising tools to support students [10,11]. Mental health apps also expand service capacity, improve availability and access to support, and reduce stigma, cost, time, and travel-related barriers for students [12-14]. Mental health apps are especially promising for students because they use smartphones frequently and are open to using apps for health-related reasons [15,16]. Research examining the effectiveness and efficacy of mental health apps among students shows that apps can improve well-being and reduce stress, depression, and anxiety [17,18].

### The JoyPop App

**Figure 1.**
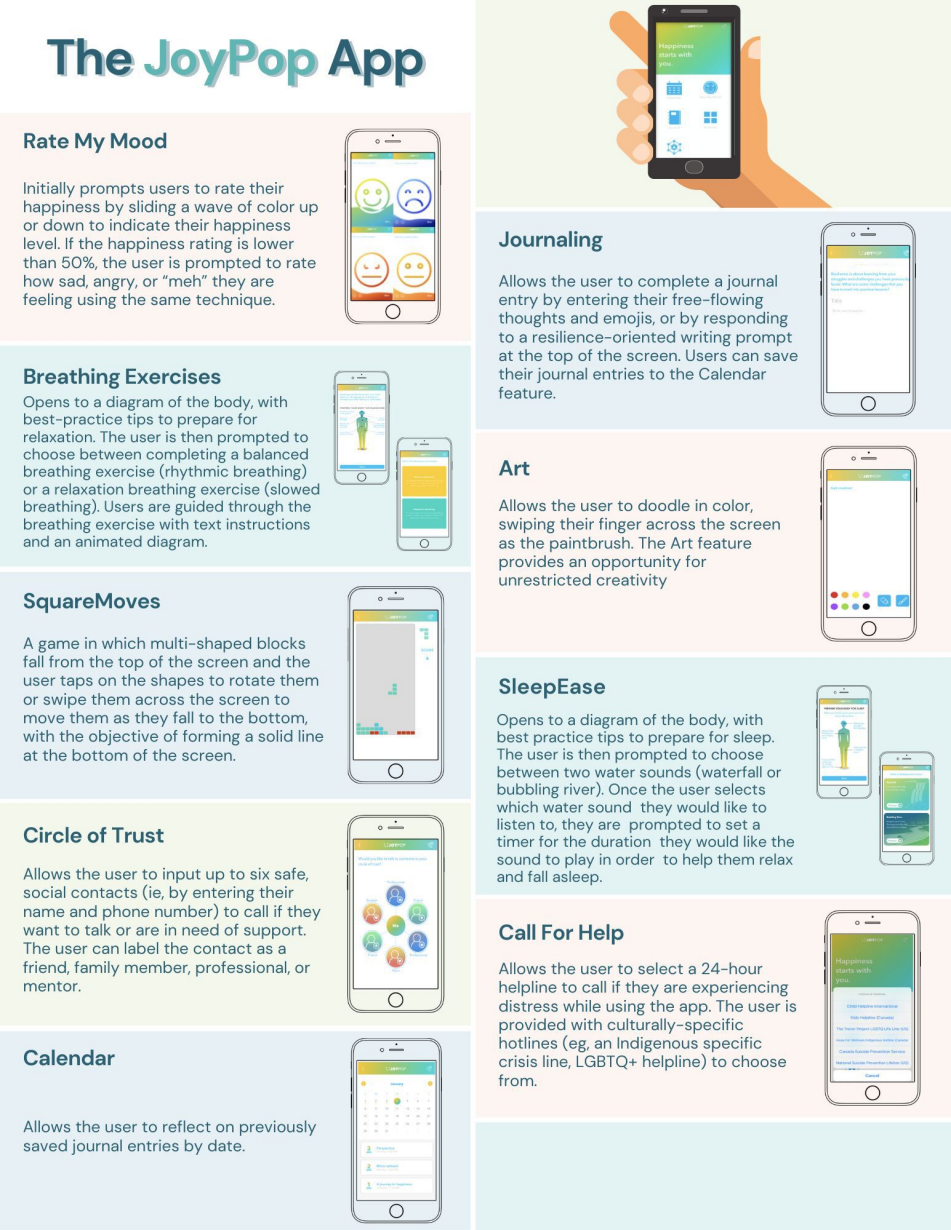
Summary and highlights of features in the JoyPop app. LGBTQ+: lesbian, gay, bisexual, transgender, and queer.

The JoyPop app is a mental health app designed to improve resilience through evidence-based features that promote emotion regulation and adaptive coping among youths and emerging adults [19,20]. The JoyPop app was co-designed by youths, researchers, and service providers [20]. The app’s transdiagnostic focus makes it a valuable addition to usual mental health supports and applicable to a diverse range of students [21,22]. The app is particularly relevant to students as many are undergoing critical life transitions, and timely interventions that promote resilience can have a long-lasting positive impact on well-being and mental health [23,24]. Details of each feature are provided in Figure 1, and the rationale for the inclusion of each feature is documented elsewhere [19,25].

There is an increasing multimethod evidence base supporting the effectiveness and value of the JoyPop app as a complement to mental health services across diverse users in clinical and nonclinical settings [19,25-29]. Among students, using the JoyPop app is associated with improved emotion regulation, improved depressive symptoms, and reduced stress [19,29]. Additionally, students perceive that the app improves emotion regulation and facilitates opportunities to implement adaptive coping skills [28]. Multiple randomized controlled trials (with students more generally [30] and with youths waiting for mental health services [31]) evaluating the app’s effectiveness in improving emotion regulation and mental health outcomes are ongoing.

Although evidence of a mental health app’s effectiveness is essential, it does not guarantee users will engage with or enjoy their experience with an app [15,32]. Student engagement and long-term uptake of mental health apps are typically low [33-35] and can reduce the overall positive outcomes associated with app use [32,36]. Engagement, safety, and overall utility are affected by how usable an app is perceived to be and a user’s perspective on the quality of an app [15,34,36]. Despite this, the majority of mental health apps available in major marketplaces have not undergone usability and quality evaluations [35,37,38].

### Usability

Usability is defined as a “quality attribute that assesses how easy interfaces are to use” [39]. Evaluating mental health app usability can reveal factors (eg, functionality, feature usefulness, layout, and readability) that reduce engagement and use [35,36]. Mental health app usability studies show that students value mental health apps with a convenient and intuitive design, customization, anonymity, privacy, peer involvement, games, and options to access professional help [40,41].

Most studies assessing usability include measures designed for general technologies [42,43], such as the System Usability Scale [44]. Applying general technology usability measures to mental health apps can provide some valuable information, but they miss identifying factors unique to mobile health apps, such as limited computation power, security and privacy challenges, and characteristics associated with small screens and portable devices [43,45]. Measures designed specifically for apps, such as the Mobile Application Usability Questionnaire (MAUQ) [43], are rarely used [42,46]. Usability evaluations with validated and standardized measures developed for apps are recommended [43,46].

### Quality

Evaluating the quality (eg, effectiveness, credibility, reliability, and safety) of mental health apps is also essential. Users are more likely to use a mental health app if it is provided by a credible source, positively impacts their mental health, and has evidence of safety and effectiveness [32]. Without proof of quality, users may rely on subjective star ratings, which can be inconsistent with an app’s actual quality and clinical utility [47-49]. Claims of mental health app quality are not consistently assessed by regulatory bodies [49,50], and most apps lack evidence of quality and effectiveness, clinical input from professionals, and evidence-based features [51,52]. Poor-quality mental health apps can therefore pose significant risks to users, such as misdiagnosis, unknown side effects, and physical and mental harm [53].

Several resources are now available to locate, assess, and regulate the quality of mental health apps, such as app rating guidelines and platforms [37,54,55]. For example, the Mobile Health App Database (MHAD) rating platform provides quality ratings for a wide variety of mental health apps to help guide users and health care providers in selecting quality apps [54,56]. The Mobile Application Rating Scale (MARS) [49] is the most popular app rating guideline [42] and allows experts in mental health to rate apps based on quality indicators from health-related fields [49]. The user version of the Mobile Application Rating Scale (uMARS) is also available to help consumers without training and expertise to rate the quality of mobile health apps [55].

### This Study

In this study, we aimed to (1) evaluate the usability and quality of the JoyPop app and (2) test the relative predictive importance of usability and quality on usage intentions, compared to other user characteristics. We used questionnaires explicitly designed to evaluate mobile health apps [42,46] to ensure that important facets of usability and quality unique to these apps were captured [43,57]. We chose age, gender, neuroticism, and agreeableness as relevant user characteristics based on past research highlighting their influence on mental health app use [58-61]. Because of the extensive literature highlighting the importance of usability and quality in mental health app use [32,37,45], we hypothesized that usability and quality would significantly predict JoyPop usage intentions over and above the other user characteristics.

## Methods

### Design

We used a 1-week prospective design to ensure students had sufficient time to use the app thereby facilitating more informed app evaluations.

### Ethical Considerations

The study was reviewed and approved by the Lakehead University research ethics board (Romeo #1470023). All participants provided informed consent before beginning the study, were under no obligation to participate, and were free to withdraw at any time without penalty. All data were deidentified. We compensated participants for their time via cash or e-transfer or bonus points toward an eligible psychology course for completing part 1 and part 3 of the study (CAD $10 [US $7.28] or 1 bonus point for part 1, CAD $20 [US $14.57] or 1.5 bonus points for part 3).

### Participants and Procedure

Participants were eligible for the study if they were students at Lakehead University who spoke and read fluently in English. We recruited students throughout the fall (2023) and winter (2024) terms (N=183). To increase the representativeness of our sample, we used multiple recruitment methods (eg, posters and class emails), had no exclusionary criteria based on student characteristics (eg, year of study), and provided refurbished iPhones with the JoyPop app for students who did not have a suitable device of their own. The study had 3 parts. In part 1, students attended an orientation session where they provided informed consent, learned about downloading and using the app, and completed preapp measures (ie, demographics and personality traits). In part 2, the research team reminded participants (via text or email) to use the app twice daily for 1 week. In part 3, participants were emailed or texted a link to complete postapp measures (ie, usability and quality).

### Measures

#### Demographics

To describe the characteristics of the sample, we captured participant demographics (13 items) along with students’ experience with mental health apps (5 items) by adapting items from prior questionnaires assessing technology experience [62,63]. We used the age and gender of participants as control variables in our analysis.

#### The Big Five Aspects Scale

We used the Big Five Aspects Scale (BFAS) [64] to gather information on participant personality traits. The BFAS consists of 100 items rated on a 5-point Likert scale ranging from 1 (strongly disagree) to 5 (strongly agree). Participants rate their level of agreement with how well brief statements generally describe how they are. The BFAS provides scores for the traditional Big Five domains and 2 aspects for each domain. We used scores for the neuroticism and agreeableness domains as control variables in our analysis. The BFAS shows excellent internal consistency and test-retest reliability in postsecondary samples [64-66]. The internal consistency of domains used in this study was excellent (neuroticism, α=.9; agreeableness, α=.82).

#### MAUQ

The MAUQ [43] assesses the usability of mobile health apps. We used the 18-item standalone patient version of the MAUQ in this study as it best suits the context and design of the JoyPop app. Overall usability includes 3 subscales: ease of use (5 items), interface and satisfaction (7 items), and usefulness (6 items). Participants rate their level of agreement with brief statements on a 7-point Likert scale ranging from 1 (strongly disagree) to 7 (strongly agree). We calculated the overall usability score by computing the mean across all items. We derived subscale scores by computing the mean of scores for relevant subscale items. Higher scores indicate greater usability. Consistent with prior research [67,68], we used descriptors based on quartiles derived from a maximum possible score of 7 on the MAUQ scales to facilitate reporting and interpreting results: 0‐1.75 (poor), 1.76‐3.5 (moderate), 3.51‐5.25 (good), and 5.26‐7 (very good). The MAUQ has adequate psychometric properties across samples, including among postsecondary students [43]. The internal consistency of the overall scale score and subscale scores in this study was strong (overall scale, α=.92; ease of use, α=.79; interface and satisfaction, α=.86; usefulness, α=.85).

#### uMARS

The uMARS is a simple and reliable tool for end users to comprehensively evaluate mobile health app quality via indicators of objective and subjective quality, and perceived impact on a target health behavior [55]. We used the 27-item uMARS to evaluate the quality of the JoyPop app from the user’s perspective. The 16-item “objective quality” scale of the uMARS allows users to rate app quality based on indicators derived from research and expert and clinician recommendations on a 5-point Likert scale ranging from 1 (inadequate) to 5 (excellent). The “objective quality” scale has 4 subscales: engagement (5 items), functionality (4 items), aesthetics (3 items), and information (4 items). We derived the overall and subscale scores by calculating the mean of relevant items (items rated as N/A were omitted). Based on item ratings and past research [69,70], we used descriptors for ranges of scores: 0‐1 (inadequate), 1‐2 (poor), 2‐3 (acceptable), 3‐4 (good), and 4‐5 (excellent), to facilitate the interpretation of results and comparisons with other mental health apps. The overall scale and subscale scores have good psychometric properties [55]. The internal consistencies in this study were good (objective quality, α=.89; engagement, α=.77; functionality, α=.75; aesthetics, α=.78; information, α=.79).

The 4-item “subjective quality” subscale of the uMARS allows users to rate different indicators (eg, star ratings, whether it is worth recommending) of a mobile health app’s subjective quality on a 5-point Likert scale ranging from 1 to 5 (anchors vary across questions). The 6-item “perceived impact” subscale of the uMARS allows users to rate its impact on user knowledge, awareness, intention to change, help-seeking, behavior change, and attitudes on a 5-point Likert scale ranging from 1 (strongly disagree) to 5 (strongly agree). This subscale was designed so that the wording of the items could be adapted to fit the function (eg, to impact mental health) of an app. We calculated the subjective quality and perceived impact subscale scores by calculating the mean of the relevant items (higher scores indicate stronger quality and impact). The subjective quality scale and perceived impact scale have good psychometric properties [55]. The internal consistency in this study was good (subjective quality, α=.79; perceived impact, α=.93).

#### Usage Intentions

We used an item (“How many times do you think you would use the JoyPop app in the next 12 months if it was relevant to you”) from the subjective quality scale on the uMARS to assess participants’ intentions to use the app in the future (usage intentions). Participants responded on a 5-point scale ranging from 1 (none) to 5 (>50 times). (Although we tracked the number of days participants used the app across the study period, we did not include this as our primary app use outcome variable as there was insufficient variation; that is, the majority of participants used the app every day; see descriptive results below.)

#### Statistical Analysis

We used SPSS (version 29, IBM Corporation) for the analyses. We calculated and examined the descriptive statistics for demographics and personality traits to characterize the sample. We calculated descriptive statistics for each scale and item of the MAUQ and uMARS to evaluate the usability and quality of the JoyPop app. To test our hypotheses that usability and quality would predict app use over and above other user characteristics, we conducted 2 multiple regression analyses. The first model included overall usability as a predictor; the second model included overall objective quality as a predictor. Both models included age, gender, agreeableness, and neuroticism as covariates. We included all predictors together for each model because our focus was to examine the relative importance of quality and usability in predicting use.

## Results

### User Characteristics

We present the characteristics of the sample in Table 1. On average, participants were aged 22.6 (SD 7.08; range 16‐56) years. Most participants were women (150/183, 82%), White (100/183, 54.6%) or Black (33/183, 18%), full-time students (156/183, 85.2%), and in their first (81/183, 44.3%) or second (54/183, 29.5%) year of university. The majority of participants reported no current or past use of mental health apps (148/183, 80.9%), and most did not currently have a mental health app installed on their phones/devices (158/183, 86.3%). Among participants who used mental health apps in the past (35/183, 19.1%), most used them for 0‐6 months (115/183, 62.8%). Descriptive statistics of relevant personality traits (ie, neuroticism: mean 3.18, SD 0.63; agreeableness: mean 4, SD 0.42) were consistent with prior research among students [64,66]. Most participants used the app every day of the study period (mean 4.69, SD 0.81).

**Table 1.** User characteristics (N=183).

	Value, n (%)
Gender	
Women	150 (82)
Men	33 (18)
Ethnicity	
White	100 (54.6)
Black	33 (18)
South Asian	20 (10.9)
Indigenous	10 (5.5)
Southeast Asian	7 (3.8)
Other (eg, Middle Eastern, South American, and Asian Canadian)	13 (7.1)
Country of birth	
Canada	116 (63.4)
Nigeria	26 (14.2)
India	14 (7.7)
Pakistan	4 (2.2)
Philippines	3 (1.6)
Other (eg, Italy, Barbados, Vietnam, Sri Lanka, Uganda, and China)	20 (10.9)
Program	
Nursing	66 (36.1)
Psychology	62 (33.9)
Education	10 (5.5)
Social work	7 (3.8)
Computer science	7 (3.8)
Biology	7 (3.8)
Other (eg, kinesiology, business, outdoor recreation, and political science)	24 (13.1)
Year of university	
1	81 (44.3)
2	54 (29.5)
3	26 (14.2)
4	19 (10.4)
>5	3 (1.6)

### Usability

Participants rated the JoyPop app’s overall usability as “very good” (mean 5.63, SD 0.85; range 2.22‐7), and 69.9% (128/183) of the sample had scores in the “very good” range. Participants rated the ease of using the app as “very good” (mean 6.37, SD 0.67; range 3.2‐7), and 92.3% (169/183) of the sample reported scores in the “very good” range. Participants rated the interface and satisfaction with the app as “very good” (mean 5.7, SD 0.99; range 1.57‐7), and 68.9% (126/183) had scores within the “very good” range. Participants rated the usefulness of the app as “good” (mean 4.93, SD 1.17; range 1.5‐7), with 42.1% (77/183) of scores in the “good” range and 38.3% (70/183) of scores in the “very good” range. We found that all item scores (see Table 2) on the ease of use and interface and satisfaction subscales fell within the “very good” range, and all item scores on the usefulness subscale were in the “good” range.

**Table 2.** Means and SDs of MAUQ^a^ subscale items.

Subscale items	Mean (SD)
Ease of use	
1. The app was easy to use.	6.58 (0.66)
2. It was easy for me to learn to use the app.	6.64 (0.55)
3. The navigation was consistent when moving between screens.	6.37 (0.94)
4. The interface of the app allowed me to use all the functions (such as entering information, responding to reminders, and viewing information) offered by the app.	6.32 (1.04)
5. Whenever I made a mistake using the app, I could recover easily and quickly.	5.96 (1.20)
Interface and satisfaction	
6. I like the interface of the app.	5.75 (1.23)
7. The information in the app was well organized, so I could easily find the information needed.	6.29 (0.92)
8. The app adequately acknowledged and provided information to let me know the progress of my action.	5.50 (1.45)
9. I feel comfortable using this app in social settings.	5.84 (1.24)
10. The amount of time involved in using this app has been fitting for me.	5.59 (1.42)
11. I would use this app again.	5.42 (1.50)
12. Overall, I am satisfied with this app.	5.55 (1.50)
Usefulness	
13. The app would be useful for my health and well-being.	5.19 (1.58)
14. The app improved my access to health care services.	4.63 (1.62)
15. The app helped me manage my health effectively.	4.52 (1.55)
16. This app has all the functions and capabilities I expected it to have.	4.85 (1.71)
17. I could use the app even when the internet connection was poor or not available.	5.16 (1.31)
18. This app provided an acceptable way to receive health care services, such as accessing educational materials, tracking my own activities, and performing self-assessments.	5.22 (1.45)

a MAUQ: Mobile Application Usability Questionnaire.

### Quality

Participants rated the overall objective quality of the app as excellent (mean 4.06, SD 0.54; range 1.96‐5), with 60.7% (111/183) of participants rating app quality as “excellent.” Participants rated the app’s functionality (mean 4.47, SD 0.52; range 2.75‐5; 136/183, 74.3% in the “excellent range”), aesthetics (mean 4.17, SD 0.67; range 1‐5; 101/183, 55.2% “excellent”; 41/183, 21.4% “good”), and information (range 1‐5; 107/183, 58.5% “excellent”; 39/183, 21.3% “good”) as “excellent.” Participants rated the app’s engagement as “good” (range 1‐5; 74/183, 40.4% “good”; 34/183, 18.6% “excellent”).

Regarding subjective quality (see Table 3), we found that many participants (144/183, 78.7%) would recommend the app to others. For our measure of app use, which asked how often participants would use the app in the next 12 months, 24% (44/183) reported 3‐10 times, 42.1% (77/183) reported 10‐50 times, and 19.1% (35/183) reported >50 times. Most participants (132/183, 72.1%) did not want to pay for the app. The most frequent star rating of the app was 4 stars (92/183, 50.3%), followed by 3 stars (62/183, 33.9%).

**Table 3.** Means, SDs, and frequencies of “subjective quality” item responses (N=183).

Item and response options	Responses, n (%)	Responses, mean (SD)
Would you recommend the JoyPop app to people who might benefit from it?		3.69 (1.22)
Not at all; I would not recommend this app to anyone	7 (3.8)	
There are very few people I would recommend this app to	32 (17.5)	
Maybe, there are several people I would recommend this app to	33 (18)	
There are many people I would recommend this app to	49 (26.8)	
Definitely, I would recommend this app to everyone	62 (33.9)	
How many times do you think you would use the JoyPop app in the next 12 months if it was relevant to you?		3.55 (1.61)
None	19 (10.4)	
1 to 2	8 (4.4)	
3 to 10	44 (24)	
10 to 50	77 (42.1)	
>50	35 (19.1)	
Would you pay for this app?		1.9 (0.98)
Definitely not	82 (44.8)	
Probably not	50 (27.3)	
Unsure	40 (21.9)	
Probably yes	9 (4.9)	
Definitely yes	2 (1.1)	
What is your overall (star) rating of the app?		3.54 (0.80)
One	3 (1.6)	
Two	13 (7.1)	
Three	62 (33.9)	
Four	92 (50.3)	
Five	13 (7.1)	

The overall perceived impact of the app on participants’ mental health and coping skills (see Table 4) was moderate (mean 3.48, SD 0.88; range 1‐5). Most participants rated the app as having a moderate impact (55/183, 30.1% of scores between ≥3 and <4) or a strong impact (45/183, 24.6% of scores ≥4). Many participants agreed or strongly agreed that the app had a positive impact on their awareness (107/183, 58.5%), behaviors (103/183, 56.3%), knowledge and understanding (103/183, 56.3%), intentions and motivation (106/183, 57.9%), and attitudes (100/183, 54.6%) related to mental health and coping skills. Most agreed or strongly agreed that the app had a helpful impact on their willingness to seek further help for mental health and coping skills if needed (115/183, 62.8%).

**Table 4.** Descriptive statistics for perceived impact subscale items (N=183).

Items	Response option frequencies, n (%)					Response, mean (SD)
	Strongly disagree	Disagree	Neither agree nor disagree	Agree	Strongly agree	
Increased my awareness of the importance of addressing mental health and coping skills	7 (3.8)	28 (15.3)	41 (22.4)	78 (42.6)	29 (15.8)	3.51 (1.05)
Increased my knowledge and understanding of mental health and coping skills	10 (5.5)	26 (14.2)	44 (24)	82 (44.8)	21 (11.5)	3.42 (1.04)
Changed my attitudes toward improving mental health and coping skills	9 (4.9)	32 (17.5)	42 (23)	79 (43.1)	21 (11.5)	3.39 (1.06)
Increased my intentions and motivation to address mental health and coping skills	7 (3.8)	26 (14.2)	44 (24)	80 (43.7)	26 (14.2)	3.49 (1.02)
Would encourage me to seek further help to address mental health and coping skills	6 (3.3)	20 (10.9)	42 (23)	81 (44.2)	34 (18.6)	3.64 (1.01)
Will improve my mental health and coping skills	10 (5.5)	19 (10.4)	51 (27.9)	86 (47)	17 (9.3)	3.44 (0.99)
Total score	—^a^	—	—	—	—	3.48 (0.88)

a Not applicable.

### Usability, Quality, and Usage Intentions

We present the results of each regression model examining the relative predictive importance of usability and quality on usage intentions in Table 5. We found evidence of heteroskedasticity for age, so we used robust standard errors to obtain unbiased estimates of the regression coefficients. Our first regression model tested the predictive importance of usability on usage intentions. Age (β=.15; *P*=.008) and gender (β=.12; *P*=.04) were both significant and positive predictors of usage intentions. Neuroticism and agreeableness were not significant predictors of usage intentions. In line with our hypothesis, usability significantly and positively predicted usage intentions over and above other user characteristics, and usability was the strongest predictor in the model (*F*_5,177_=15.69; *P*<.001; *R*^2^_adjusted_=0.29; β=.56; *P*<.001). Our second regression model tested the predictive importance of quality on usage intentions. Age was a significant and positive predictor of usage intentions (β=.17; *P*=.008). Gender, neuroticism, and agreeableness were not significant predictors of usage intentions. Consistent with our hypothesis, quality significantly and positively predicted usage intentions over and above other user characteristics and was the strongest predictor in the model (*F*_5,177_=12.58; *P*<.001; *R*^2^_adjusted_=0.24; β=.52; *P*<.001).

**Table 5.** Multiple regression analysis predicting intention to use the JoyPop app in the future.

Predictors	B^a^	Robust SE	β	*P* value
Model 1				
Constant	0.14	1.05	—^b^	.90
Gender	–0.38	0.18	.12	.04
Age	0.03	0.01	.15	.01
Neuroticism	–0.02	0.13	–.01	.86
Agreeableness	–0.26	0.18	–.10	.15
Usability	0.76	0.10	.56	<.001
Model 2				
Constant	0.26	1.07	—	.81
Gender	–0.36	0.20	.12	.07
Age	0.03	0.01	.17	.01
Neuroticism	–0.06	0.14	–.03	.66
Agreeableness	–0.34	0.18	–.12	.06
Quality	1.12	0.17	.52	<.001

a Unstandardized coefficient.

b Not applicable.

## Discussion

### Usability and Quality of the JoyPop App

We evaluated the usability and quality of the JoyPop app and assessed their impact on intentions to use the app in the future after controlling for other user characteristics. Participants rated the overall usability as “very good” and all indicators comprising usability subscales were in the “good” to “very good” range. The cumulative evidence strongly supports that JoyPop app users are comfortable and satisfied with using it, find it easy to learn and use, and view it as a helpful tool to support mental health and well-being in different contexts (eg, clinical and academic settings) [25-28]. Usability ratings for the JoyPop app from this study are also similar to ratings of other popular mental health apps [71]. These findings will support organizations and consumers who are considering offering or using the JoyPop app.

Participants rated the JoyPop app’s overall objective quality, functionality, aesthetics, and quality of information as “excellent” and the app’s engagement as “good.” Results are consistent with past research on the JoyPop app, highlighting it as an engaging, high-quality mental health app with strong functionality, visual design, and helpful evidence-based content [25-28]. Results also indicate that the JoyPop app falls within the upper ranges for objective quality when compared to popular and research-supported mental health apps (eg, Calm, PTSD Coach, SuperBetter, and Destressify) rated by experts [54,72]. For example, across approximately 50 mental health apps rated using the MARS, the average objective quality was 3.54 (range 1.63‐4.75) [54]. Among 19 popular research-supported mental health apps, the average objective quality score was 3.52 (SD 0.71), and average subscale scores were 3.98 (SD 0.82) for engagement, 3.42 (SD 0.80) for functionality, 3.23 (SD 0.90) for aesthetics, and 3.47 (SD 0.69) for information [72].

Response frequencies to items on the subjective quality scale also support the quality of the JoyPop app. Although these are subjective ratings, their importance is highlighted by the consistency between subjective consumer ratings of mental health apps and objective expert reviews of mental health apps [72]. Subjective quality results also showed that most participants would not pay for the app. This is not surprising, as research consistently shows that people are less likely to use apps that have associated costs [32,73]. Taken together, the high objective and subjective quality ratings of the JoyPop app across indicators of mobile health app quality, combined with its quality ratings compared to mental health apps reviewed by experts, strongly support its safety, credibility, helpfulness, and use for students. Future research will benefit from having experts evaluate the quality of the JoyPop app (eg, using the MARS) to validate this study’s findings and by compiling end-user quality evaluations of mental health apps to facilitate comparisons.

Finally, more than half of the participants perceived that the JoyPop app positively impacted their mental health and coping skills in all areas queried. This finding further supports the overall quality of the app. The broad range of areas impacted by the JoyPop app is consistent with past research demonstrating its effectiveness and benefits among diverse samples of users and supports its overall ability to deliver users an effective experience [19,25-29]. Results from rigorously controlled trials examining effectiveness are still needed. However, results on the app’s perceived impact on mental health and coping skills can delineate whether future outcomes are meaningful and impactful, provide researchers with an understanding of the potential mechanisms and reasons why the app might be effective, and inform outcome measures that can be assessed in rigorously controlled trials (eg, measures of help-seeking and coping skills).

### Influence of Usability and Quality on Usage Intentions

In our regression models, overall usability and objective quality were the strongest predictors of usage intentions and predicted usage intentions over and above age, gender, neuroticism, and agreeableness. Although we did not objectively measure app usage (eg, number of days used), behavioral intentions are a strong predictor of actual behavior, and user intentions are consistently shown to predict the use of digital health technologies [74-76]. Consequently, our results align with research demonstrating the substantial impact of quality and usability on engagement, long-term uptake, and use of mental health apps [32,35,37]. The importance of usability and quality in predicting participants’ intentions to use the JoyPop app in the future, along with insight into indicators influencing its quality and usability, provides integral information for JoyPop and other mental health app development teams in optimal app design to improve user experience. An iterative process can be used in which development teams have users rate the usability and quality of the app, determine specific indicators requiring improvement, adapt the app based on these indicators, and then re-evaluate the usability and quality among similar populations.

### Limitations

This study has some important limitations. First, we used a convenience sample primarily comprised of women, which may reduce the generalizability of results to men and the broader student population. However, the demographics and diversity of our sample help support the generalizability of the results to students because this study’s demographics are similar to those found among students across Ontario [77]. Second, in our study, we requested that participants use the app twice a day over 7 days. This likely contributed to the limited variability in the number of days participants used the app and necessitated our use of a subjective measure of usage intention instead of actual use. To better capture objective usage metrics, studies conducted over longer periods without prompting participants to use the app are needed to establish the relative predictive importance of usability and quality on app use [57].

### Conclusions

Mental health apps are promising tools for students’ mental health. This study evaluated the usability and quality of a mental health app (JoyPop) designed to improve resilience while examining the relative predictive importance of usability and quality in predicting intentions to use the app in the future. Participants rated the JoyPop app as having “very good” usability. Participants rated the app as having high overall quality because of its “excellent” objective quality, good subjective quality, and helpful impact on their mental health and coping skills. After accounting for user characteristics, usability and quality were the strongest predictors of participants’ intentions to use the JoyPop app in the future. Findings facilitate consistency and comparisons of mental health app usability and quality and contribute to the growing evidence base supporting the JoyPop app as an engaging and high-quality mental health app that can support students.

## Abbreviations

BFAS: Big Five Aspects Scale
MARS: Mobile Application Rating Scale
MAUQ: Mobile Application Usability Questionnaire
MHAD: Mobile Health App Database
uMARS: user version of the Mobile Application Rating Scale
